# Impact of Inadequate Number of Lymph Nodes Examined on Survival in Stage II Colon Cancer

**DOI:** 10.3389/fonc.2021.736678

**Published:** 2021-09-20

**Authors:** Qi Wu, Zhiyuan Zhang, Yijiao Chen, Jiang Chang, Yudong Jiang, Dexiang Zhu, Ye Wei

**Affiliations:** Department of General Surgery, Zhongshan Hospital, Fudan University, Shanghai, China

**Keywords:** number, lymph nodes, survival, stage II colon cancer, retrospective

## Abstract

**Background:**

Inadequate number of lymph nodes examined was not uncommon. We aimed to assess the clinical role of inadequate number of lymph nodes examined in stage II colon cancer.

**Methods:**

The cancer data used in our study were obtained from the SEER (Surveillance, Epidemiology and End Results) program. Using the chi-square test, all the variables obtained in our study were compared based on whether patients had enough (≥12) lymph nodes examined. Kaplan–Meier analysis was used for overall survival (OS) analysis, and log-rank test was applied to compare different N stages with the total number of lymph nodes examined. Multivariate analysis was carried out by creating a Cox proportional hazard model to assess the prognostic roles of different variables.

**Results:**

In total, 80,296 stage II/III colon cancer patients were recruited for our study. N0 stage with <8 lymph nodes examined would present with a worse prognosis compared to N1 stage (5-year OS rates, 51.6% vs. 57.1%, *p* < 0.001). Multivariate analyses indicated that OS of N0 stage with <8 lymph nodes examined was similar to that of N1 stage after adjusting for other recognized prognostic factors [hazard ratios (HRs) = 1.051, 95% confidence intervals (CIs) = 1.014–1.090, *p* = 0.018].

**Conclusions:**

N0 stage with less than eight lymph nodes examined in stage II colon cancer presented with no better OS compared to that of N1 stage. Stage II colon cancer with less than eight lymph nodes examined needed to be given greater emphasis in clinical practice.

## Introduction

Colon adenocarcinoma was ranked as one of the most frequent malignant tumors worldwide (1). The tumor node metastasis (TNM) cancer staging system proposed by the American Joint Committee on Cancer (AJCC) has been widely used to guide treatment plans and evaluate prognosis of colon cancer patients. Based on this staging system, the most obvious difference between stage II and stage III colon cancer was the presence of pathological lymph node metastasis.

Recommended by the American College of Pathologists (ACP) and the American Joint Committee on Cancer (AJCC), the pathological examination of lymph nodes should be at least 12 in the surgical specimen, which has become the general consensus to ensure accurate nodal staging in colon cancer patients (2, 3). It was reported by researchers that the least number of lymph nodes examined was 13 to identify more than 90% local lymph node metastasis (4). In spite of that, inadequate number of lymph nodes examined was not uncommon. Inadequate number of lymph nodes examined might lead to inaccurate assessment for lymph node involvement, which would misguide clinical treatment decisions and adversely affect patient outcomes (5, 6).

It was previously reported in a big retrospective study that the number of lymph nodes examined ranged between 5.5 and 21.3 lymph nodes, and the average lymph node harvest was 11.7, which was less than the recommended minimum lymph nodes examined (7). In the present study, using the data from a big cancer database with long‐term follow-up, we aimed to evaluate the prognostic value of inadequate number of lymph nodes examined in stage II colon cancer.

## Materials and Methods

### Patients

The cancer data used in our study were obtained from the SEER (Surveillance, Epidemiology and End Results) program. Established by the National Cancer Institute, it collected and updated de-identified patient information regarding cancer incidence, clinicopathological characteristics, treatment modalities, survival, etc.

The patient flow diagram is shown in **Supplementary Figure S1**. From 2004 to 2010, patients diagnosed with colon cancer were identified using the NCI SEER*Stat software (version 8.3.5). All patients meeting the following criteria were eligible for inclusion in the present study: (1) with surgical resection; (2) with active follow-up; (3) without distant metastasis; (4) with positive histological confirmation; (5) T3 or T4; (6) with exact number of nodes examined; and (7) adenocarcinoma histology. Besides stage II colon cancer, we have also recruited stage III (stage T3–4 with lymph node metastasis) patients to compare the prognosis of inadequate lymph nodes examined and pathological lymph node positivity.

The following demographic and clinicopathologic characteristics including tumor grade, gender, the time of diagnosis, TNM stage, race, tumor histology, and the receipt of chemotherapy were subsequently required from this cancer database. For N stage, it has been divided into different subgroups according to the examined number of lymph nodes to assess the clinical role of inadequate number of lymph nodes examined in stage II colon cancer.

### Statistical Analyses

In the present study, overall survival (OS) was considered to be the outcome of interest. OS was defined as the time from the date of diagnosis with colon cancer to time of death. In the first, using the chi-square test, all the variables obtained in our study were compared based on whether patients had enough (≥12) lymph nodes examined.

Kaplan–Meier analysis was used for OS analysis, and the log-rank test was applied to compare different node stages with the number of lymph nodes examined in total. According to the above variables, multivariate analysis was carried out by creating a Cox proportional hazard model to assess the clinical role of different variables with 95% confidence intervals (CIs) and hazard ratios (HRs). Analyses were performed by using IBM SPSS Statistics for Windows, Version 25.0. (IBM Corp., Armonk, NY). In our analyses, only tests producing a two-sided *p* < 0.05 would reach statistical significance.

## Results

### Patients’ Demographics and Clinical Characteristics

In total, 80,296 stage II/III colon cancer patients were recruited for our study; demographic and clinicopathologic characteristics including tumor grade, gender, the time of diagnosis, TNM stage, race, tumor histology, and the receipt of chemotherapy were then acquired from this cancer database. Besides stage II colon cancer, we have also recruited stage III (stage T3–4 with lymph node metastasis) patients to compare the prognosis of inadequate lymph nodes examined and pathological lymph node positivity.

**Figure 1:**
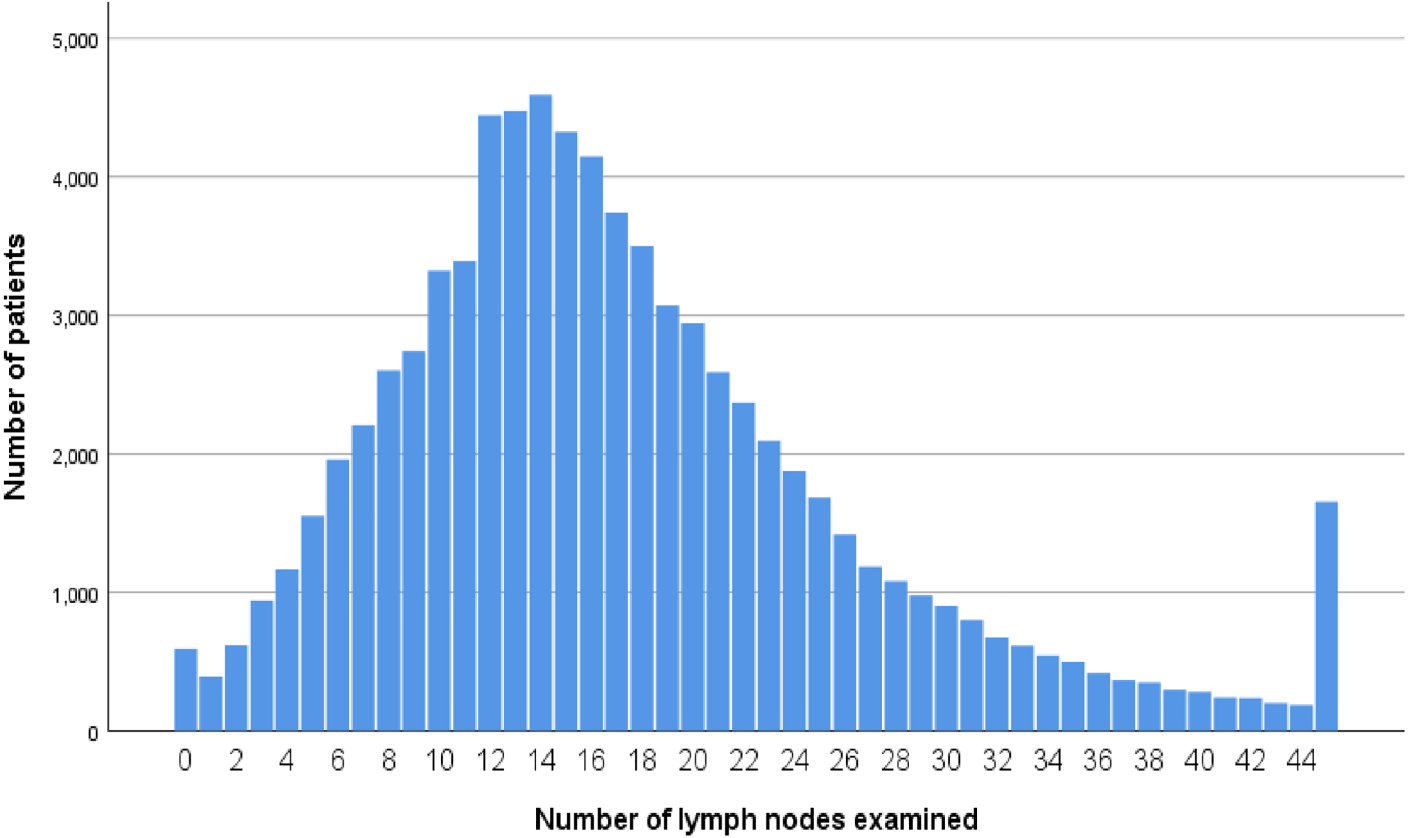
Distribution of numbers of lymph node retrieved.

In total, male patients accounted for 48.0% (38,537). As shown in **Figure 1**, the median number of lymph nodes examined was 16, and there was a total of 45,146 (56.2%) deaths at end of the follow-up. The demographic and clinicopathological characteristics of colon cancer patients were listed (**Table 1**). In all, 58,799 patients (73.2%) had more than 12 lymph nodes examined. The results of chi-square test also indicated that white patients (*p* = 0.013), ≤75 years old (*p* < 0.001), T3 stage (*p* = 0.034), stage N2 (*p* < 0.001), poor/anaplastic grade (*p* < 0.001), female (*p* < 0.001), mucinous/signet ring cell histology (*p* < 0.001), and later year of diagnosis (*p* < 0.001) were more likely to have enough (≥12) lymph nodes examined.

**Table 1:** Demographics and clinical characteristics.

Characteristics	No. of lymph nodes examined (%)		*p*-value
	<12 (*n* = 21,497)	≥12 (*n* = 58,799)	
**Race**			0.013
** White**	17,340 (80.7)	47,882 (81.4)	
** Black**	2,547 (11.8)	6,536 (11.1)	
** Other**	1,610 (7.5)	4381 (7.5)	
**Age at diagnosis**			<0.001
** ≤75 years**	11,660 (54.2)	36,608 (62.3)	
** >75 years**	9,837 (45.8)	22,191 (37.7)	
**T stage**			0.034
** T3**	17,951 (83.5)	49,464 (84.1)	
** T4**	3,546 (16.5)	9,335 (15.9)	
**N stage**			<0.001
** N0**	12,789 (59.5)	31,748 (54.0)	
** N1**	6,374 (29.7)	16,079 (27.3)	
** N2**	2,334 (10.9)	10,972 (18.7)	
**Tumor grade**			<0.001
** High/Moderate**	16,700 (77.7)	43,430 (73.9)	
** Poor/Anaplastic**	4,328 (20.1)	14,318 (24.4)	
** Unknown**	469 (2.2)	1,051 (1.8)	
**Sex**			<0.001
** Male**	10,575 (49.2)	27,962 (47.6)	
** Female**	10,922 (50.8)	30,837 (52.4)	
**Histology**			<0.001
** Adenocarcinoma**	19,287 (89.7)	51,692 (87.9)	
** Mucinous/signet ring cell**	2,210 (10.3)	7,107 (12.1)	
**Year of diagnosis**			<0.001
** 2004–2007**	15,878 (73.9)	30,885 (52.5)	
** 2008–2010**	5,619 (26.1)	27,914 (47.5)	
**Chemotherapy**			<0.001
** No**	15,267 (71.0)	38,086 (64.8)	
** Yes**	6,230 (29.0)	20,713 (35.2)	

### Inadequate Number of Lymph Nodes Examined Had Poor Prognosis

**Figure 2:**
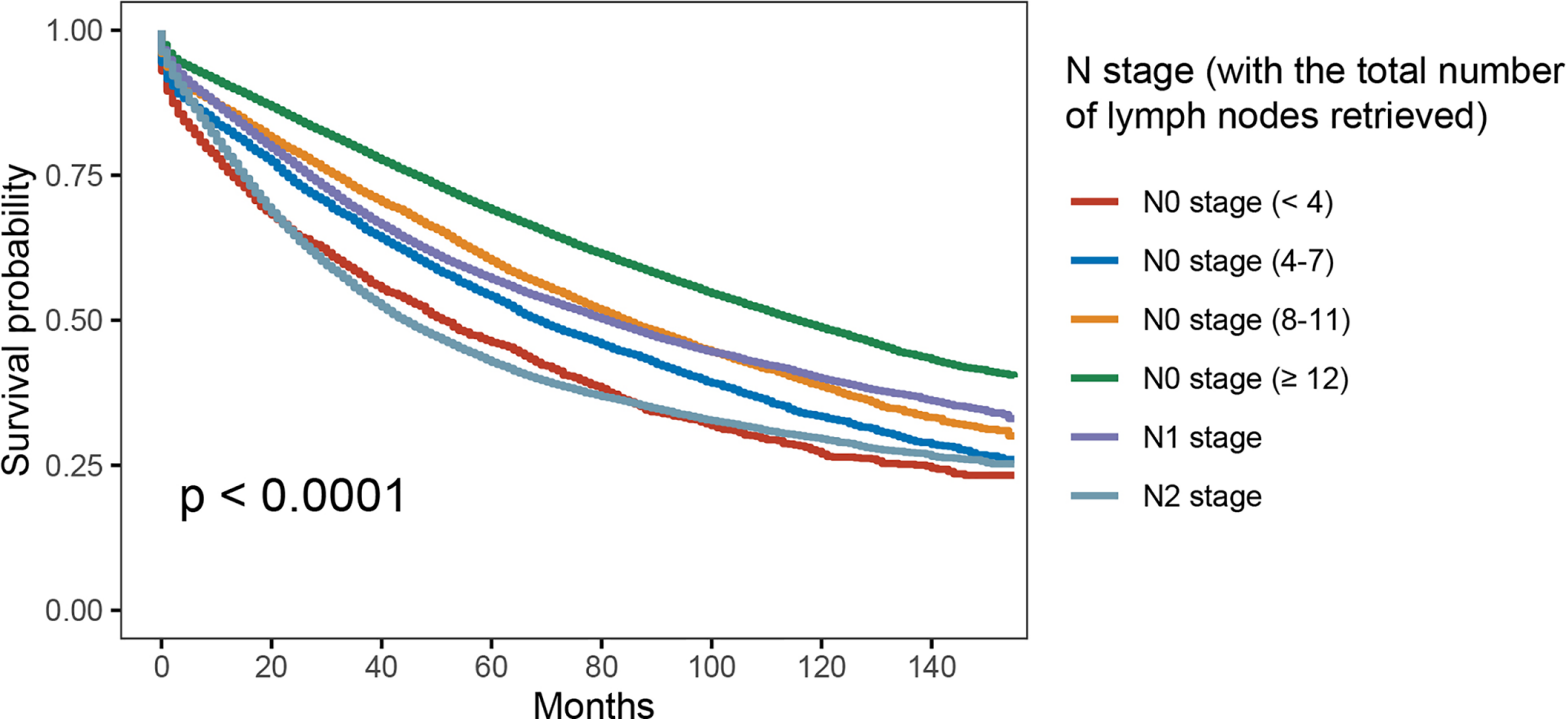
Kaplan–Meier survival curves for colon cancer patients according to number of lymph node retrieved.

In **Figure 2**, patients would present with worse OS as the number of lymph nodes examined decreased in node-negative cases—<4 lymph nodes (5-year OS rate = 46.2%) was more likely to have decreased OS compared with <8 lymph nodes (5-year OS rate = 54.1%; *p* < 0.001), <8 lymph nodes was more likely to have decreased OS compared with <12 lymph nodes (5-year OS rate= 60.2%; *p* < 0.001), and < 12 lymph nodes was more likely to have worse OS compared with more than 12 lymph nodes examined (5-year OS rate = 69.0%; *p* < 0.001). Moreover, results of Kaplan–Meier analyses indicated that <8 lymph nodes with node negativity was even more likely to have decreased OS compared with N1 stage (5-year OS rates, 54.1% *vs.* 57.1%, *p* < 0.001).

In addition, multivariate analyses were conducted based on the Cox proportional hazards model with the recognized prognostic factors included, which showed that patients would present with worse OS as the number of lymph nodes examined decreased in node-negative cases. Moreover, patients with N1 stage were prone to having better OS compared to N0 stage with <4 lymph nodes examined (HR = 0.909, 95%CI = 0.860–0.962, *p* = 0.001; **Table 2**).

**Table 2:** Multivariate analyses on overall survival in colon cancer.

Characteristics	Overall survival		
	HR (95% CI)	SE	*p*-value
**Race**			<0.001
** White**	Reference		
** Black**	1.196 (1.161–1.232)	0.015	<0.001
** Other**	0.780 (0.750–0.812)	0.020	<0.001
**Age at diagnosis**			<0.001
** ≤75 years**	Reference		
** >75 years**	2.524 (2.474–2.576)	0.010	
**Histology**			0.005
** Adenocarcinoma**	Reference		
** Mucinous/signet ring cell**	1.042 (1.013–1.072)	0.015	
**T stage**			<0.001
** T3**	Reference		
** T4**	1.650 (1.611–1.689)	0.012	
**N stage**			<0.001
** N0 stage (<4 lymph nodes)**	Reference		
** N0 stage (<8 lymph nodes)**	0.810 (0.759–0.864)	0.033	<0.001
** N0 stage (<12 lymph nodes)**	0.690 (0.649–0.733)	0.031	<0.001
** N0 stage (≥12 lymph nodes)**	0.541 (0.512–0.572)	0.028	<0.001
** N1 stage**	0.909 (0.860–0.962)	0.029	0.001
** N2 stage**	1.389 (1.311–1.471)	0.029	<0.001
**Tumor grade**			<0.001
** High/Moderate**	Reference		
** Poor/Anaplastic**	1.147 (1.122–1.172)	0.011	<0.001
** Unknown**	1.131 (1.059–1.208)	0.033	<0.001
**Sex**			<0.001
** Male**	Reference		
** Female**	0.839 (0.824–0.855)	0.010	
**Year of diagnosis**			0.547
** 2004–2007**	Reference		
** 2008–2010**	1.006 (0.986–1.026)	0.010	
**Chemotherapy**			<0.001
** No**	Reference		
** Yes**	0.586 (0.572–0.600)	0.012	

### The Comparison of OS Between Node Positivity and Inadequate Lymph Nodes Examined

To better evaluate the clinical role of inadequate number of lymph nodes examined in stage II colon cancer, we then compared the OS difference between node-positive patients and node-negative patients (with inadequate lymph nodes examined).

**Figure 3:**
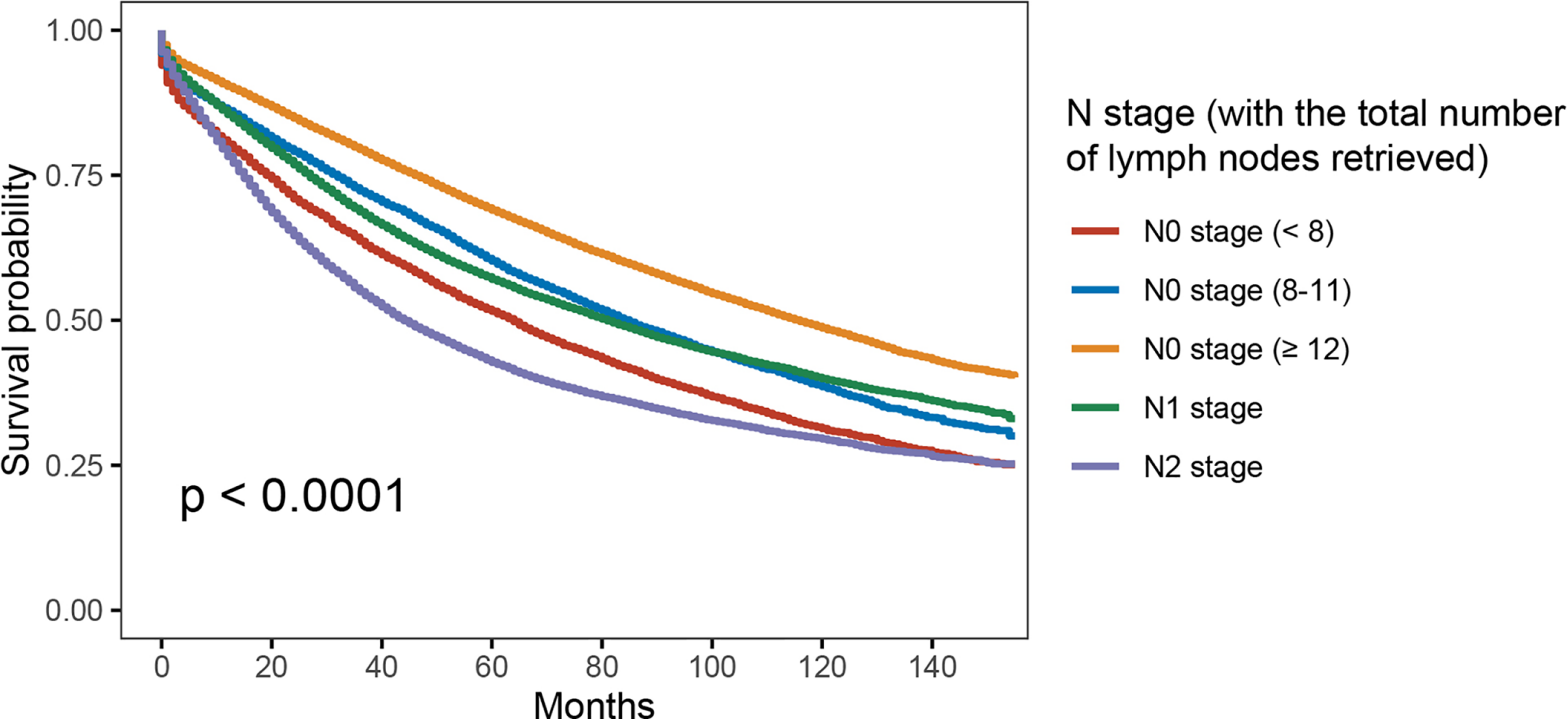
Kaplan–Meier survival curves for colon cancer patients according to the number of lymph node retrieved (with the combination of less than four lymph nodes and less than eight lymph nodes examined together).

As shown in **Figure 3**, with the combination of less than four lymph nodes and less than eight lymph nodes examined together in node-negative patients, it was found that N0 stage with <8 lymph nodes examined would present with worse OS compared to stage N1 (5-year OS rates, 51.6% vs. 57.1%, *p* < 0.001). The results also showed that the survival difference between N0 stage with <12 lymph nodes examined and N1 stage was not statistically significant (*p* = 0.918). Then, multivariate analyses were conducted based on the Cox proportional hazard model with the recognized prognostic factors included; the results indicated that the OS of N0 stage with <8 lymph nodes was similar to that of N1 stage after adjusting for other recognized prognostic factors (HR = 1.051, 95%CI = 1.014–1.090, *p* = 0.018; **Table 3**).

**Table 3:** Multivariate analyses on overall survival in colon cancer (with the combination of less than four lymph nodes and less than eight lymph nodes retrieved together).

Characteristics	Overall survival		
	HR (95% CI)	SE	*p*-value
**Race**			<0.001
** White**	Reference		
** Black**	1.195 (1.161–1.231)	0.015	<0.001
** Other**	0.780 (0.750–0.812)	0.020	<0.001
**Age at diagnosis**			<0.001
** ≤75 years**	Reference		
** >75 years**	2.524 (2.474–2.575)	0.010	
**Histology**			0.005
** Adenocarcinoma**	Reference		
** Mucinous/signet ring cell**	1.041 (1.012–1.072)	0.015	
**T stage**			<0.001
** T3**	Reference		
** T4**	1.651 (1.612–1.690)	0.012	
**N stage**			<0.001
** N0 stage (<8 lymph nodes)**	Reference		
** N0 stage (<12 lymph nodes)**	0.798 (0.764–0.833)	0.022	<0.001
** N0 stage (≥12 lymph nodes)**	0.626 (0.605–0.648)	0.018	<0.001
** N1 stage**	1.051 (1.014–1.090)	0.018	0.006
** N2 stage**	1.606 (1.545–1.669)	0.020	<0.001
**Tumor grade**			<0.001
** High/Moderate**	Reference		
** Poor/Anaplastic**	1.146 (1.122–1.172)	0.011	<0.001
** Unknown**	1.136 (1.064–1.213)	0.033	<0.001
**Sex**			<0.001
** Male**	Reference		
** Female**	0.839 (0.824–0.855)	0.010	
**Year of diagnosis**			0.508
** 2004–2007**	Reference		
** 2008–2010**	1.007 (0.987–1.027)	0.010	
**Chemotherapy**			<0.001
** No**	Reference		
** Yes**	0.586 (0.572–0.600)	0.012	

## Discussion

The high and increasing incidence of colon cancer around the world made the related studies a hot topic in recent years (8–11). In this study, we aimed to evaluate the clinical role of inadequate number of lymph nodes examined in stage II colon cancer, with comparison of node positivity in stage III colon cancer.

With a median follow-up time of 75 months, 80,296 colon cancer patients were enrolled in the whole cohort. Therefore, this was a large retrospective study with long-term follow-up, and it has added more credibility to our main findings. The median number of lymph nodes examined in total was 16, which exceeded the recommended number of lymph nodes examined according to the clinical recommendations of AJCC and ACP. Our results have revealed that up to 26.8% of all the colon cancer patients did not have adequate retrieval of the recommended number of lymph nodes. However, adequate lymphadenectomy has been suggested for colon cancer, and recent studies showed that increased number of lymph nodes examined for pathological identification would contribute to improved OS in colon cancer, indicating the necessity of finding ways to increase the number of lymph nodes examined (12–15).

To the best of our knowledge, the present population-based study was the largest one that has addressed the clinical role of inadequate lymph node examined in stage II colon cancer. It was found that white race, younger age, later tumor stage, advanced tumor grade, female, mucinous/signet ring cell histology, and later year of diagnosis were prone to being associated with enough (≥12) lymph nodes removed. Previous studies also reported that the length of the bowel resection and the location of ligation of the blood vessels (such as a higher ligation in a complete excision) could affect the number of lymph nodes retrieved (16). Apart from that, several other factors such as the extent and the technique of surgical dissection, physiological factors, and the pathologist’s work experience and technical level were also found to exert an effect on the lymph nodes examined (17–19).

Using Kaplan–Meier method, our initial analysis indicated that N0 stage with <4 lymph nodes examined would present with worse OS compared with N1 stage (46.2% *vs.* 57.1%, *p* < 0.001). Moreover, N0 stage with less than eight but more than four lymph nodes examined (5-year OS rate = 54.1%) also presented with worse OS compared with N1 stage (*p* < 0.001). In addition, multivariate analyses were conducted based on the Cox proportional hazards model with the recognized prognostic factors included; the results showed that the OS of N1 stage had 9.1% decreased risk of overall mortality compared with less than four lymph nodes in node-negative patients after adjusting for other recognized factors.

Therefore, we have combined less than four lymph nodes with more than four but less than eight lymph nodes examined together in node-negative patients. The Kaplan–Meier survival analyses indicated that N0 stage with <8 lymph nodes examined would present with worse OS compared to stage N1 (5-year OS rates, 51.6% *vs.* 57.1%, *p* < 0.001). Other recognized prognostic characteristics were then taken account into multivariate analyses, which indicated that the OS of N0 stage with <8 lymph nodes examined was similar to that of N1 stage.

In our analyses, the result that survival of N0 stage with less than eight lymph nodes examined was comparable to that of N1 stage was parallel to previous findings (3, 14, 20). As early as 2002, it was reported by Cianchi and his colleagues (20) that the 5-year survival rate of the Dukes B patients with <8 retrieved lymph nodes was similar to that of the Dukes C patients. Then, in 2005, Sarli et al. (14) recruited 745 stage II and 493 stage III colorectal cancer patients in Italy and found that patients with stage III tumor with only one to three positive lymph nodes had a similar 5-year survival rate (52.6%) compared with that of patients with stage II colon cancer with less than nine lymph nodes examined in total (51.3%).

Similarly in 2009, one British study showed that the survival of Duke’s B (stage II) colorectal cancer patients with less than nine lymph nodes examined in total was not better than that of Duke’s C (stage III) disease patients (3). In spite of that, it was noteworthy that the abovementioned three studies did not enroll enough cases to evaluate the prognostic value of inadequate number of lymph nodes examined in stage II colon cancer. Moreover, in the present study, with 80,296 patients recruited from a big cancer database, we concluded that N0 stage with less than eight lymph nodes examined in stage II colon cancer presented with similar OS compared to that of N1 stage. Stage II colon cancer with less than eight lymph nodes examined needed to be given greater emphasis in clinical practice.

As suggested by the clinical recommendations of European Society for Medical Oncology, some clinical factors such as pT4 stage, bowel obstruction or tumor perforation, lymph nodes sampling <12, lymphatic or vascular or peripheral invasion, and poorly differentiated tumor were listed as risk characteristics in stage II disease (21). Recently, some researchers have questioned the efficacy of chemotherapy in high-risk stage II colon cancer patients (22–24). In 2018, it was also reported by some American researchers that adjuvant chemotherapy was not associated with improved survival if less than 12 lymph nodes was deemed as the sole high-risk factor (25). The number of enrolled patients in the current study was sufficiently large to give us confidence that the results were high in reliability to evaluate the clinical role of inadequate number of lymph nodes examined in stage II colon cancer. The results of our analyses, together with what have been discussed above, made us believe that stage II colon cancer with less than eight lymph nodes examined needed to be given greater emphasis in clinical practice. However, it should be recognized that preoperative chemotherapy and postoperative chemotherapy were mixed up as chemotherapy and thus analyzing whether neoadjuvant treatment would have effect on the number of lymph nodes examined because of the limitation of this cancer database. Improvements in technology and research in oncology have greatly improved the prognosis of colon cancer patients in the past decade, which may have an influence on the findings of our study. Moreover, the selection bias would also exist in a study with retrospective design. Therefore, it would be interesting to carry out a prospective study to further explore the clinical role of less than eight lymph nodes examined in offering adjuvant therapy in stage II colon cancer.

## Conclusions

Our study showed that OS of N0 stage with less than eight lymph nodes examined was comparable to that of N1 stage in stage II colon cancer. Therefore, stage II colon cancer with less than 8 lymph nodes examined needed to be given greater emphasis in clinical practice. It would be interesting to carry out a prospective study to further explore the clinical role of less than eight lymph nodes examined in offering adjuvant therapy in stage II colon cancer.

## Data Availability Statement

The raw data are available from the corresponding author upon reasonable request.

## Author Contributions

DZ and YW conceived and designed the study. QW and ZZ contributed to the acquisition of data. QW, YC, JC, and YJ interpreted the data. QW wrote the first draft of the manuscript. DZ and YW reviewed and edited the manuscript. All authors contributed to the article and approved the submitted version.

## Conflict of Interest

The authors declare that the research was conducted in the absence of any commercial or financial relationships that could be construed as a potential conflict of interest.

## Publisher’s Note

All claims expressed in this article are solely those of the authors and do not necessarily represent those of their affiliated organizations, or those of the publisher, the editors and the reviewers. Any product that may be evaluated in this article, or claim that may be made by its manufacturer, is not guaranteed or endorsed by the publisher.

## Supplementary Material

Supplementary Figure 1 Patient selection flow diagram.
