# Supplementary material for: Impact of Inadequate Number of Lymph Nodes Examined on Survival in Stage II Colon Cancer
Source: Front Oncol. 2021 Sep 20;11:736678. doi: 10.3389/fonc.2021.736678 (PMC8489731; doi:10.3389/fonc.2021.736678)
Supplement: Supplementary Figure 1 — Patient selection flow diagram. [file DataSheet_1.pdf]

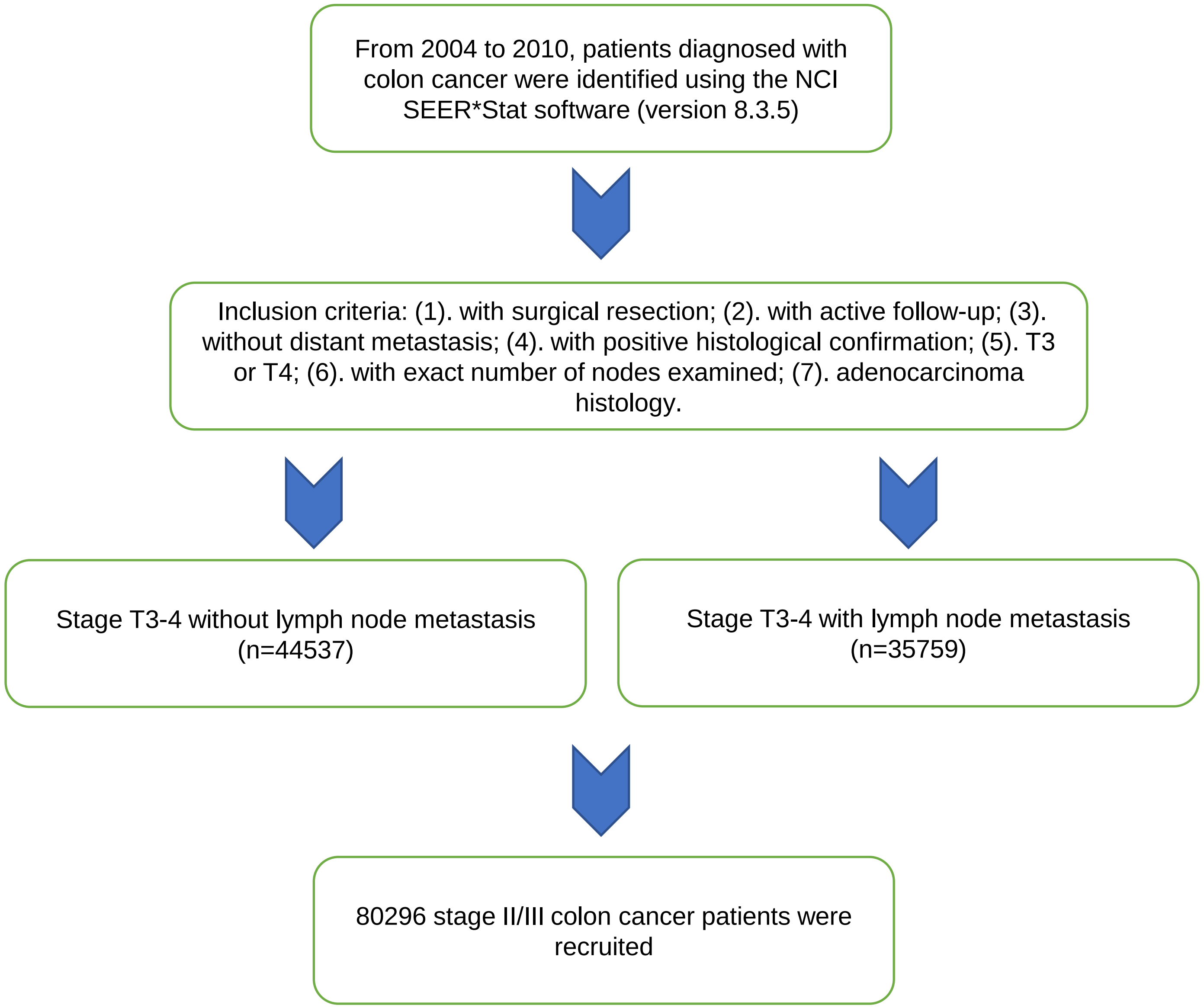
From 2004 to 2010, patients diagnosed with colon cancer were identified using the NCI SEER*Stat software (version 8.3.5)
Inclusion criteria: (1). with surgical resection; (2). with active follow-up; (3). without distant metastasis; (4). with positive histological confirmation; (5). T3 or T4; (6). with exact number of nodes examined; (7). adenocarcinoma histology.
Stage T3-4 without lymph node metastasis (n=44537)
Stage T3-4 with lymph node metastasis (n=35759)
80296 stage II/III colon cancer patients were recruited
